# Novel Compound C150 Inhibits Pancreatic Cancer Cell Epithelial-to-Mesenchymal Transition and Tumor Growth in Mice

**DOI:** 10.3389/fonc.2021.773350

**Published:** 2021-12-15

**Authors:** Tao Wang, Ping Chen, Ruochen Dong, Scott Weir, Michael Baltezor, Frank J. Schoenen, Qi Chen

**Affiliations:** ^1^ Department of Pharmacology, Toxicology and Therapeutics, University of Kansas Medical Center, Kansas City, KS, United States; ^2^ Department of Cancer Biology, University of Kansas Medical Center, Kansas City, KS, United States; ^3^ Biotechnology Innovation and Optimization Center, University of Kansas, Lawrence, KS, United States; ^4^ Higuchi Biosciences Center, University of Kansas, Lawrence, KS, United States

**Keywords:** pancreatic cancer, EMT, Snail, protein degradation, tumor growth

## Abstract

Pancreatic cancer cell epithelial-to-mesenchymal transition (EMT) is an important contributor to cell invasion and tumor progression. Therefore, targeting EMT may be beneficial for pancreatic cancer treatment. The aim of the present study was to report on the inhibitory effect of the novel compound C150 on the EMT of pancreatic cancer cells. C150 inhibited cell proliferation in multiple pancreatic cancer cells with IC_50_ values of 1-2.5 μM, while in an non-cancerous pancreatic epithelial cell line hTERT-HPNE the IC_50_ value was >12.5 μM. C150 significantly inhibited pancreatic cancer cell migration and invasion, as demonstrated by 3-dimensional cell invasion, wound healing and Boyden chamber Transwell migration-invasion assays. Moreover, C150 treatment decreased MMP-2 gene expression in PANC-1 cells and reduced MMP-2 activity in gelatin zymography assay. In an orthotopic mouse model of pancreatic cancer, C150 significantly reduced tumor growth at the dose of 15 mg/kg by intraperitoneal injection three times per week. Furthermore, C150 enhanced protein degradation of Snail, an important EMT-promoting transcription factor, and decreased the expression of the mesenchymal marker N-cadherin, while it increased the expression of the epithelial markers zonula occludens-1 and claudin-1. The findings of the present study suggested that C150 is a novel EMT inhibitor that may be promising for inhibiting pancreatic cancer growth and metastasis.

## Introduction

Pancreatic ductal adenocarcinoma (PDAC) accounts for 90% of all cases of pancreatic cancer (1) and is one of the most lethal types of cancer, with an overall 5-year survival rate of only 9% (2). Despite numerous efforts, the treatment options for PDAC remain limited, and the treatment outcomes are poor. Gemcitabine as a single agent has been used as first-line chemotherapy for almost three decades (3), but its benefits in terms of survival are limited. A recently developed combination therapy with gemcitabine and nab-paclitaxel improved the median overall survival by a few months, but was also associated with increased incidence of treatment-related toxicities (4). A non-gemcitabine combination regimen comprising oxaliplatin, irinotecan, fluorouracil and leucovorin (FOLFIRINOX) has also demonstrated a small benefit in terms of survival compared with gemcitabine, but the toxicities of this regimen are severe and have been reported to be intolerable by a significant proportion of the patients (5, 6). The high mortality rate of PDAC is largely due to early metastasis, which accounts for the poor treatment outcomes (6, 7). Therefore, identifying new anti-pancreatic cancer agents is crucial.

Emerging evidence has shown that epithelial-to-mesenchymal transition (EMT) plays an important role in PDAC cell invasion, metastasis, tumor progression and drug resistance (8, 9). The initial steps of metastasis involve the dissemination of cancer cells from the tumor bulk and invasion through the extracellular matrix (ECM), in which EMT plays a critical role (10). By initiating the EMT process, the apical-basal polarized carcinoma cells lose cell-cell adhesions and are converted to fibroblast-like mesenchymal cells, exhibiting enhanced mobility and invasiveness (11–13). In a transgenic mouse model of pancreatic cancer, EMT was shown to occur in the early stages of tumor formation and was associated with cancer cell dissemination and invasion prior to, and in parallel with, primary pancreatic tumor formation (14). Moreover, in human tissue samples of pancreatic cancer, it has been found that the epithelial marker E-cadherin was weakly expressed in poorly differentiated tumors, and the mesenchymal marker N-cadherin was mainly expressed in the invasive front (15). The EMT-promoting factor Snail was found to be highly expressed in tumor tissues compared with normal tissues (15). Snail has been reported to suppress the expression of epithelial markers, such as E-cadherin, claudins and zonula occludens (ZO)-1 (16–18), and to promote the expression of mesenchymal markers, such as fibronectin, N-cadherin and MMPs (12, 19–21). Overexpression of Snail was reported to induce EMT and promote pancreatic cancer cell invasion and metastasis in mouse models of pancreatic cancer (22). These previous findings strongly indicate the significance of EMT in promoting pancreatic cancer progression. Thus, targeting EMT may be beneficial for the treatment of this disease.

We previously established a high-throughput screening assay for the discovery of compounds that had the potential to enhance E-cadherin expression (23). Upon screening of combined libraries of ~47,000 compounds, several positive hits that could potentially inhibit EMT in pancreatic cancer cells were identified (23). The aim of the present study was to investigate one of the top hits, compound C150 (2-[2-(5-nitro-2-thienyl)vinyl]quinoline), a quinoline compound with a novel structure, for its activity in suppressing pancreatic cancer EMT and tumor progression *in vitro* and *in vivo.*

## Materials And Methods

### Cell Culture and Reagents

The human pancreatic cancer cell lines PANC-1, MIA PaCa-2, HPAF-II and BxPC-3 cells were obtained from the American Type Culture Collection. The immortalized human pancreatic duct epithelial cell line hTERT-HPNE was donated by Dr. Shrikant Anant and L3.6pl pancreatic cancer cells were donated by Dr Liang Xu at the University of Kansas. All cancer cells were cultured in recommended media with 10% FBS (cat. no. F0926; Sigma-Aldrich; Merck KGaA) and 100 U/ml penicillin/streptomycin (cat. no. 30-001-CI; Corning Life Science) and were used within 20 passages in our laboratory. The hTERT-HPNE cells were cultured with DMEM (cat. no. 10-013 CV; Corning Life Science) supplemented with 5% FBS, 1X N2 supplement (cat. no. 17502-048; Invitrogen; Thermo Fisher Scientific, Inc.), 10 ng/ml basic fibroblast growth factor (cat. no. PHG0024; Invitrogen; Thermo Fisher Scientific, Inc.) and 50 μg/ml gentamicin (cat. no. 15710-064; Gibco; Thermo Fisher Scientific, Inc.), and were used within 10 passages in our laboratory. All cells were cultured at 37˚C in a humidified cell incubator with 5% CO_2_. C150 compound was purchased from ChemBridge and stocked in DMSO. All C150 treatments were diluted in cell culture medium with a final DMSO concentration <0.1% (v/v%). All control (Ctrl) groups were treated with the same volume of medium containing DMSO (<0.1% v/v%).

### MTT Cell Viability Assay

Cells were seeded in 96-well plates at 5,000 cells/well and incubated overnight, and then treated with C150 for 48 h. MTT (20 μl of 5 mg/ml solution) was added into each well and incubated at 37°C for 4 h. The medium was then removed and 150 μl DMSO was added into each well. Absorbance was detected at 570 nm using a microplate reader (BioTek Instruments, Inc.).

### Transwell Migration and Invasion Assays

Cells were seeded (5x10^4^ cells/insert) into Transwell cell culture inserts (cat. no. 353097; Corning Life Science) coated (invasion assay) or non-coated (migration assay) with 1 mg/ml (0.1%) Matrigel (cat. no. 356237; Corning Life Science) in pure DMEM medium (cat. no. 10-013 CV; Corning Life Science) without FBS. The inserts were then placed in 24-well tissue culture plates with culture medium supplemented with 10% FBS (cat. no. F0926; Sigma-Aldrich; Merck KGaA) to serve as a chemoattractant in the wells. Treatments were added in the medium in inserts and in wells. After 24 or 48 h of treatment, the inserts were removed from the well, and cells inside the inserts were removed using cotton swab. Cells on the bottom of the insert membrane were fixed in 4% formaldehyde for 10 min, followed by 10 min staining in 0.5% crystal violet solution at room temperature. The inserts were then washed in water and left to dry before being photographed. Images of the whole insert were captured under a light microscope. The total number of cells on each insert membrane were counted using ImageJ software (Java 1.8.0_172 version for Mac OS X, National Institutes of Health).

### Wound Healing Assay

Cells were seeded in a 24-well plate at a density of 2x10^5^ cells/well and cultured to a confluent monolayer. A linear scratch was made on the monolayer using a 200-μl pipette tip. Cell debris was washed away with fresh medium. Cells were then cultured for 24 h with or without treatments. Images from 5 different areas in each well were captured at 0, 12 and 24 h under a Nikon eclipse TE 2000-U phase contrast inverted microscope at a magnification of x100. The distance between the edges of the wound was measured using ImageJ software (Java 1.8.0_172 version for Mac OS X, National Institutes of Health), and cell migration was quantified as follows:

Wound healing (%) = (distance_0 h_ - distance_24 h_)/distance_0 h_.

### Three-Dimensional (3D) Cell Invasion Assay

PANC-1 cells were seeded at a density of 8,000 cells/well in an ultra-low attachment, round-bottomed 96-well plate (cat. no. 7007; Corning Life Science) in complete growth medium and cultured for 4 days to form compact spheroids (one spheroid/well). Type I rat tail collagen (cat. no. 354236; Corning Life Science) was adjusted to neutral pH with setting solution (100 ml 10X EBSS, 2.45 g NaHCO_3_, 7.5 ml 1M NaOH and 42.5 ml H_2_O) and kept on ice. The medium was fully removed from each well, followed by the addition of 100 μl of neutral-pH collagen. The plate was then placed in a 37°C cell incubator for 30 min for collagen to solidify. A total of 100 μl of complete growth medium was then added into each well and images were captured at 0 h. Treatments were added to both collagen and medium. The spheroids were treated for 72 h and images were captured using phase contrast light microscopy at a magnification of x40. Cell invasion was analyzed using ImageJ software (Java 1.8.0_172 version for Mac OS X, National Institutes of Health) by drawing an enclosed line tracing the invasion edge, encircling the total area. Then, an enclosed line was drawn along the core spheroid to encircle the core spheroid area. The invaded area was calculated by subtracting the core spheroid area from the total area. Invasion score = invaded area/core spheroid area.

### Western Blotting

Total cell lysate and tumor tissue lysate were obtained using Pierce RIPA buffer (cat. no. 89901; Thermo Fisher Scientific, Inc.) in the presence of protease and phosphatase inhibitor cocktails (cat. nos. P8340, P5726 and P0044; Sigma-Aldrich; Merck KGaA). Nuclear and cytoplasmic fraction lysis was performed according to the protocol by Pierce NE-PER Nuclear and Cytoplasmic Extraction kit (cat. no. 78833; Thermo Fisher Scientific, Inc.). Lysate protein concentration was determined using Pierce BCA protein assay (cat. no. 23225; Thermo Fisher Scientific, Inc.). The lysate was mixed with 2X Laemmli SDS loading buffer (cat. no. 161-0737; Bio-Rad Laboratories, Inc.), ran in an 8 or 10% SDS-PAGE gel and transferred onto 0.2 μm PVDF membranes (cat. no. ISEQ00010; Millipore Sigma). The membranes were blocked in 5% blocking grade milk in TBST solution (0.1% Tween-20 in 1X TBS) for 2 h at room temperature, then incubated with primary antibody at 4°C overnight in 5% BSA (cat. no. BP1605-100, Fisher Scientific)/TBST solution. Primary antibodies included rabbit anti-PARP (dilution 1:1,000, cat. no. 9542S; Cell Signaling Technology, Inc.), rabbit anti-caspase-3 (dilution 1:1,000, cat. no. 9662S; Cell Signaling Technology, Inc.), rabbit anti-vinculin (dilution 1:1,000, cat. no. 4650S; Cell Signaling Technology, Inc.), rabbit anti-Snail (dilution 1:500, cat. no. 3879S; Cell Signaling Technology, Inc.), rabbit anti-ZO-1 (dilution 1:500, cat. no. 13663S; Cell Signaling Technology, Inc.), rabbit anti-claudin-1 (dilution 1:500, cat. no. 13995S; Cell Signaling Technology, Inc.), rabbit anti-N-cadherin (dilution 1:1,000, cat. no. 13116S; Cell Signaling Technology, Inc.), rabbit anti-Histone-3 (dilution 1:2,000, cat. no. 4499S; Cell Signaling Technology, Inc.), rabbit anti-α-tubulin (dilution 1:2,000, cat. no. 2144S; Cell Signaling Technology, Inc.), mouse anti-β-actin (dilution 1:2,000, cat. no. 3700S; Cell Signaling Technology, Inc.), rabbit anti-cofilin (dilution 1:5,000, cat. no. 5175T; Cell Signaling Technology, Inc.), rabbit anti-p-cofilin (dilution 1:1,000, cat. no. 3313T; Cell Signaling Technology, Inc.), mouse anti-proliferating cell nuclear antigen (PCNA) (dilution 1:1,000, cat. no. sc-25280; Santa Cruz Biotechnology, Inc.), mouse anti-MMP-2 (dilution 1:200, cat. no. sc-53630; Santa Cruz Biotechnology, Inc.). Following incubation with HRP-linked secondary antibodies (dilution 1:5,000; anti-mouse cat. no. 7076S; anti-rabbit cat. no. 7074S; Cell Signaling Technology, Inc.) for 2 h at room temperature, the Pierce ECL plus reagent (cat. no. 32132; Thermo Fisher Scientific, Inc.) was used to establish the blots. All western blotting experiments were repeated at least three times. Western blot band density was analyzed and quantified using ImageJ software (Java 1.8.0_172 version for Mac OS X, National Institutes of Health).

### Reverse Transcription-Quantitative (RT-q) PCR Analysis

Total RNA was extracted from the cells using TRIzol^®^ reagent (cat. no. AM9738; Invitrogen; Thermo Fisher Scientific, Inc.) according to the manufacturer’s protocol. cDNA synthesis was carried out with 1 μg of total RNA using OneScript cDNA Synthesis Kit (cat. no. G234; Applied Biological Materials). cDNA was then diluted at 1:5 in nuclease-free H_2_O. RT-qPCR was performed using BioRad iQ iCycler detection system with One-Step BrightGreen reagents (cat. no. MasterMix-S; Applied Biological Materials) according to the manufacturer’s protocol. Each reaction was carried out in a volume of 10 μl with 5 μl 2X BrightGreen qPCR MasterMix, 0.6 μl forward and reverse primer mix (10 μM), 2 μl diluted cDNA and 2.4 μl nuclease-free H_2_O. All qPCR reactions were run under the following cycling conditions: Enzyme activation at 95°C for 10 min, 40 cycles of denaturation (95°C for 15 sec), and annealing/extension (60°C for 60 sec). Melting curves were detected at 55-95°C with 0.5°C increments. Three independent experiments were performed, and reactions were carried out in triplicates for each experiment. Gene expression was quantified using the 2^-^δδ^Cq^ method (24) with GAPDH as the internal control. The primer sequences were as follows: Snail (forward: 5’-TCGGAAGCCTAACTACAGCGA-3’, reverse: 5’-AGATGAGCATTGGCAGCGAG-3’); MMP-2 (forward: 5’-TAGCTGCTGGCTCACTGTGT-3’, reverse: 5’-CTTCAGCACAAACAGGTTGC-3’); GAPDH (forward: 5’-CCAGGTGGTCTCCTCTGACTTCAACA-3’, reverse: 5’-AGGGTCTCTCTCTTCCTCTTGTGCTC-3’).

### MMP Gelatin Zymography

PANC-1 cells were seeded in a 6-well plate at 1x 10^5^ cells/well. The next day, cells were treated with C150 in 1 ml FBS-free DMEM medium (cat. no. 10-013 CV; Corning Life Science) for 24 h. The supernatant was collected and processed with Pierce BCA protein assay (cat. no. 23225; Thermo Fisher Scientific, Inc.) to determine the protein concentration. The supernatant was then mixed with 5X non-reducing loading buffer (cat. no. 39001; Thermo Fisher Scientific, Inc.). An equal amount (10 μg) of total protein in each sample was run in an 10% SDS-PAGE gel containing 0.2% gelatin (Sigma-Aldrich; Merck KGaA). The gel was renatured in renaturing/washing buffer [1 M Tris (pH 8.0), 1 M CaCl_2_, 2.5% TritonX-100] for 1 h at room temperature, equilibrated in incubation buffer [1 M Tris (pH 8.0), 1 M CaCl_2_] for 10 min at room temperature, and incubated in incubation buffer at 37°C for 24 h. The gel was then stained with Coomassie Brilliant Blue R-250 (cat. no. 1610436; Bio-Rad Laboratories, Inc.) followed by de-staining in de-staining solution (40% methanol, 10% acetic acid, 50% H_2_O) at room temperature until clear bands were visible. Clear bands representing MMP enzyme activity were analyzed and quantified using ImageJ software (Java 1.8.0_172 version for Mac OS X, National Institutes of Health).

### Snail Overexpression in PANC-1 Cells

PANC-1 cells were seeded in a 6-well plate at 2x 10^5^ cells/well and cultured for 24 hours before transfection. The cells were then transfected with 125 ng pCMV-Flag-Snail plasmid (Addgene, Inc.) or 125 ng empty vector plasmid (Addgene, Inc.) using Lipofectamine™ 3000 (cat. no. L3000015; Invitrogen; Thermo Fisher Scientific, Inc.) according to the manufacturer’s protocol. At 48 h after transfection, cells were collected and seeded for wound healing assay as described earlier. Snail overexpression was confirmed by western blotting.

### Immunohistochemistry

Paraffin-embedded 5-μm tissue sections were deparaffinized and hydrolyzed through sequential xylene, 100% ethanol, 95% ethanol, 75% ethanol and H_2_O. Antigen retrieval was carried out by submerging the slides in citrate buffer (pH 6.0) for 30 min at 95°C. Immunohistochemistry staining was performed according to the protocol provided with the Abcam IHC detection kit (cat. no. ab64264; Abcam). Briefly, following antigen retrieval, the slides were blocked in the protein blocking solution for 1 h, then incubated with primary anti-PCNA antibody (dilution 1:100, cat. no. sc-25280; Santa Cruz Biotechnology, Inc.) in TBST (1X TBS with 0.1% Tween-20) solution overnight at 4°C. After washing in TBST solution, the slides were incubated in H_2_O_2_ blocking solution for 15 min, then incubated with biotinylated secondary antibody for 1 h, followed by 30-min incubation with streptavidin-peroxidase solution. DAB chromogen was then applied to the slides to develop the staining. After immunostaining, the slides were counterstained in Mayer’s hematoxylin solution for 30 sec. The slides were observed under a light microscope at a magnification of x200. Images were captured from at least 5 different fields per sample. Data were analyzed using ImageJ (Fiji Version) by subtracting the hematoxylin staining color to reveal only DAB staining color. Then, DAB-positive stained cells were counted for each image.

### Cell Cycle Analysis

A total of 5x 10^5^ PANC-1 cells were seeded in a 60-mm petri dish. The next day, the cells were treated with C150 for 48 h. Cells were collected, washed with 1X PBS and fixed in 70% ethanol at -20°C overnight. After 70% ethanol fixation, cells were washed once with 1X PBS and stained in PI staining solution (20 µg/ml PI in 1X PBS solution with 200 µg/ml RNase A and 0.1% Triton X-100) at 37°C for 15 min. Cell cycle distribution with flow cytometry (BD LSR II, BD Biosciences).

### Orthotopic Pancreatic Cancer Mouse Model

#### Dose Determination

All animal experiments followed an Animal Care and Use Protocol (2018–2443) approved by the Institutional Animal Care and Use Committee at the University of Kansas Medical Center. The acute maximal tolerance dose of C150 in mice was determined using 6 mice (Strain: BALB/cJ, 3 female, 3 male) starting with a one-time intraperitoneal (IP) dose of 20 mg/kg (in 20% H_2_O + 20% DMSO + 60% PEG-400). All mice appeared lethargic and were sacrificed by CO_2_ euthanasia 2 h after the injection. The dose was reduced to 10 mg/kg in another set of 6 mice, and no signs of toxicity were observed. Then, the dose was raised to 15 mg/kg and the mice were monitored for 7 days, during which time no signs of toxicity were observed. To further detect tolerance for sub-chronic administration of C150 at 15 mg/kg, 6 mice were administered C150 at 15 mg/kg daily for 5 consecutive days. No signs of toxicity were observed, indicating good tolerance. Based on the data and establishing a margin of safety, the treatment regimen was determined to be 15 mg/kg, 3 times per week by IP injection.

#### Tumor Treatment

Luciferase-labeled PANC-1 (PANC-1-Luc) cells were established by the Preclinical Proof of Concept Core Laboratory at the University of Kansas Medical Center (Kansas City, USA). During tumor inoculation, 6-week-old female nude mice (Hsd: Athymic Nude-Foxn1^nu^, Harlan) were anesthetized by isoflurane inhalation (5% for induction of anesthesia and 2% for maintenance). A subcostal laparotomy was performed to expose the pancreas. A total of 4x10^5^ PANC-1-Luc cells suspended in 50 μl of cold 1X PBS were injected into the tail of the pancreas. The wound was then sealed with wound clips. A total of 19 mice were inoculated. Two weeks after tumor cell inoculation, the mice were administered 150 mg/ml of D-luciferin (GoldBio) by IP injection and imaged by the IVIS imaging system (PerkinElmer, Inc, Waltham, MA). Mice were grouped into vehicle (n=10) and treatment (n=9) groups based on imaging to ensure even tumor burdens in each group. Treatments then commenced with 15 mg/kg of C150 or vehicle (20% H_2_O + 20% DMSO + 60% PEG-400), by IP injection, 3 times per week. Treatments were continued for 6 weeks. Mice were imaged weekly to monitor tumor growth. Following sacrifice, the tumors were removed, weighed, and fixed in 4% formaldehyde or frozen at -80°C for future analysis.

### Toxicity Monitoring

Body weight was monitored weekly. Body condition score (BCS) and clinical signs of pain and distress were used as indicators of toxicity. Mice reaching BCS2 or less, or exhibiting any of the signs below were considered to display signs of toxicity: Guarding, reduced movement, abnormal appearance (hunched), restlessness, circling, convulsion or blindness, rapid or labored breathing, hemorrhage, flaccid or spastic paralysis, inability to ambulate, recumbency or mutilation.

### Statistical Analysis

All results are presented as mean ± SD unless stated otherwise. Statistical analysis was performed using unpaired Student’s t-test or Mann-Whitney U test for two-group comparisons. One-way ANOVA with Tukey’s *post hoc* test was used for multi-group comparisons. P<0.05 was considered to indicate statistically significant differences.

## Results

### C150 Inhibits Proliferation in Multiple Pancreatic Cancer Cell Lines

**Figure 1:**
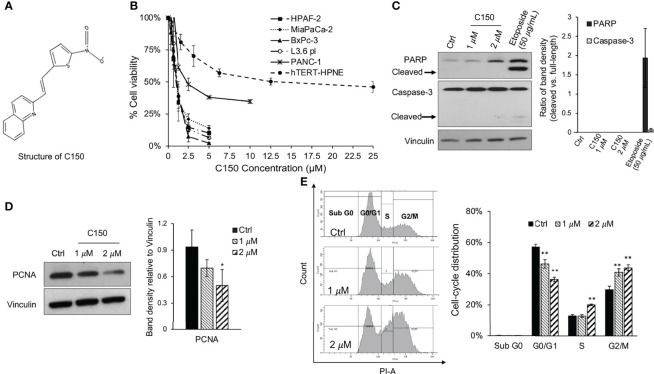
C150 inhibits pancreatic cancer cell proliferation. **(A)** Structure of C150. **(B)** Cell viability upon C150 treatment. The human pancreatic cancer cell lines HPAF-2, MIA PaCa-2, BxPC-3, L3.6pl and PANC-1, and the immortalized normal human pancreatic ductal epithelial cell hTERT-HPNE, were incubated with increasing concentrations of C150 for 48 h. Cell viability was analyzed by MTT assay and normalized to untreated controls. **(C, D)** Western blot analysis of **(C)** apoptosis markers and **(D)** PCNA. PANC-1 cells were treated with DMSO (Ctrl) or C150 (1 and 2 μM) or etoposide (50 μg/ml) for 48 h. Vinculin was blotted as the loading control. **(C)** Bar graph shows the ratio of cleaved PARP band density to full PARP band density and the ratio of cleaved caspase-3 to full Caspase-3 band density. **(D)** Bar graph shows the PCNA band density relative to vinculin. **(E)** Cell cycle analysis. PANC-1 cells were treated with DMSO (Ctrl) or C150 (1 and 2 µM) for 48 h. Cells were then collected, fixed in 70% ethanol, stained with PI and examined by flow cytometry. Left panel shows representative figures of cell cycle distribution. Bar graph shows the percentage distribution of each phase. Data represent mean ± SD of at least three independent experiments in all graphs. ^*^P*<*0.05, ^**^P*<*0.01 (vs. Ctrl) by one-way ANOVA-Tukey’s test. C150, 2-[2-(5-nitro-2-thienyl)vinyl]quinoline; PCNA, proliferating cell nuclear antigen; Ctrl, control.

The effects of C150 (**Figure 1A**) on cell viability were examined in a panel of human pancreatic cancer cell lines (PANC-1, BxPC-3, MIA PaCa-2, HPAF-II, L3.6pl) and an immortalized human pancreatic duct epithelial cell line, hTERT-HPNE. Treatment for 48 h resulted in a significant decrease in cell viability in all tested pancreatic cancer cells. The 50% inhibitory concentrations (IC_50_) were ~1 μM for BxPC-3, MIA PaCa-2, HPAF-II and L3.6pl cells, and ~2.5 μM for PANC-1 cells. The non-cancerous hTERT-HPNE cells were markedly more resistant to C150 treatment, with an IC_50_ of ~12.5 μM and the inhibitory effect was not notably enhanced when drug concentration increased further (**Figure 1B**). These data indicated a preferential inhibitory effect of C150 towards the viability of pancreatic cancer cells vs. normal cells. To examine whether the inhibition of cell viability was due to apoptosis or suppression of proliferation, the expression of two apoptosis markers, PARP and caspase-3, were examined by western blotting in PANC-1 cells treated with C150 (1 and 2 μM for 48 h). Etoposide (50 μg/ml) was used as a positive control. Compared with etoposide, which induced extensive PARP cleavage and caspase-3 cleavage, C150 did not induce PARP or caspase-3 cleavage (**Figure 1C**), indicating that C150 did not promote cell apoptosis. By contrast, the cell proliferation marker, PCNA, was significantly decreased with the same C150 treatments (**Figure 1D**). Cell cycle analysis revealed a G2/M phase arrest induced by C150 treatment, and no sub-G0 cells were detected (**Figure 1E**). These data suggested that C150 inhibited pancreatic cancer cell proliferation rather than inducing apoptosis.

### C150 Inhibits Migration and Invasion in Pancreatic Cancer Cells

**Figure 2:**
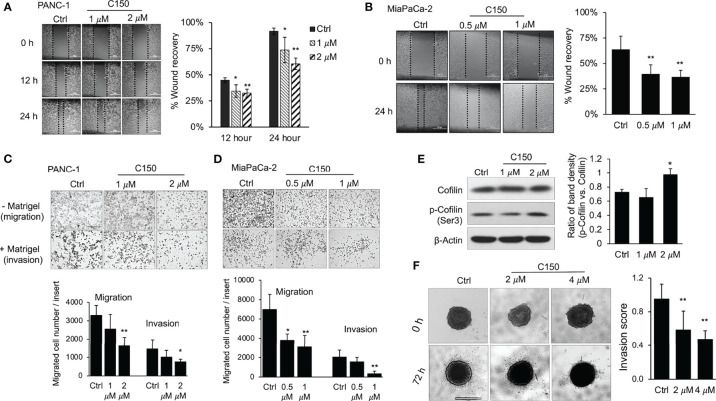
C150 inhibits pancreatic cancer cell migration and invasion. **(A, B)** Wound healing assay. Monolayer of PANC-1 **(A)** cells were treated with DMSO (Ctrl) or C150 (1 and 2 μM) for 12 and 24 h after creating the wound. MIA PaCa-2 cells **(B)** were treated with DMSO (Ctrl) or C150 (0.5 and 1 µM) for 24 h after creating the wound. Scale bar, 500 µm. Bar graph shows percentage of wound recovery (mean ± SD of 4 repeats). **(C, D)** Transwell migration and invasion assays of **(C)** PANC-1 and **(D)** MIA PaCa-2 cells. Boyden chamber Transwell inserts were either uncoated (migration) or coated (invasion) with 0.1% Matrigel. Cells were treated with DMSO (Ctrl) or C150 (1 and 2 μM for 48 h for PANC-1 cells; and 0.5 and 1 μM for 24 h for MIA PaCa-2 cells). Bar graphs show the number of migrated or invaded cells per insert (mean ± SD of two independent experiments, each performed in triplicates). Scale bar, 500 µm. **(E)** Western blots of cofilin and p-cofilin. PANC-1 cells were treated with DMSO (Ctrl) or C150 (1 and 2 µM) for 24 h. Total cell lysates were subjected to immunoblotting against cofilin and p-cofilin (Ser3). β-Actin was blotted as loading control. Bar graph shows the ratio of p-Cofilin (Ser3) band density to Cofilin band density (mean ± SD of three independent experiments). **(F)** 3D invasion assay. PANC-1 cell spheroids were grown in collagen and treated with DMSO (Ctrl) or C150 (2 and 4 μM) for 72 h. Scale bar, 1000 µm. Bar graph shows invasion score (mean ± SD of two independent experiments, each performed in eight repeats) ^*^P*<*0.05, ^**^P*<*0.01 (vs. Ctrl) by one-way ANOVA followed by Tukey’s *post hoc* test. C150, 2-[2-(5-nitro-2-thienyl)vinyl]quinoline; Ctrl, control; p-, phosphorylated.

Pancreatic cancer cell migration was first evaluated in a wound healing assay. Treatment with C150 significantly inhibited PANC-1 cell migration at 1 and 2 μM (**Figure 2A**) and MIA PaCa-2 cell migration at 0.5 and 1 µM (**Figure 2B**). Boyden chamber Transwell migration/invasion assays were then used to further assess the effects of C150 on migration (without Matrigel coating) and invasion (with Matrigel coating). After 48 h of treatment, C150 reduced the number of PANC-1 cells on the outside of the Transwell membrane in a concentration-dependent manner, with or without Matrigel coating (**Figure 2C**). The same results were further confirmed in, MIA PaCa-2 cells (**Figure 2D**). Consistently, the phosphorylation of cofilin, an actin-binding protein, was increased following treatment with 2 μM C150 in PANC-1 cells (**Figure 2E**), indicating inhibition of cytoskeleton rearrangement and decreased cell mobility (25). These data indicated that both migration and invasion were inhibited by C150 treatment.

A 3D cell invasion model was then utilized to more closely simulate the *in vivo* conditions of pancreatic cancer invasion, in which a tumor spheroid was cultured surrounded by collagen-rich ECM. PANC-1 cells were first cultured in complete growth medium in a round-bottomed ultralow-attachment 96-well plate to form compact cell spheroids. The cell spheroids were then cultured in type I collagen matrix. Upon 72 h of culture, cells around the edge of the spheroid disseminated and invaded through the surrounding collagenous ECM, forming a radial-shaped area of invasion (**Figure 2F**). C150 treatment at 2 and 4 μM significantly inhibited the invasion of PANC-1 cells in this model (**Figure 2F**). Taken together, both 2D and 3D culture data consistently demonstrated that C150 inhibited pancreatic cancer cell migration and invasion *in vitro*.

### C150 Suppresses EMT in Pancreatic Cancer Cells

**Figure 3:**
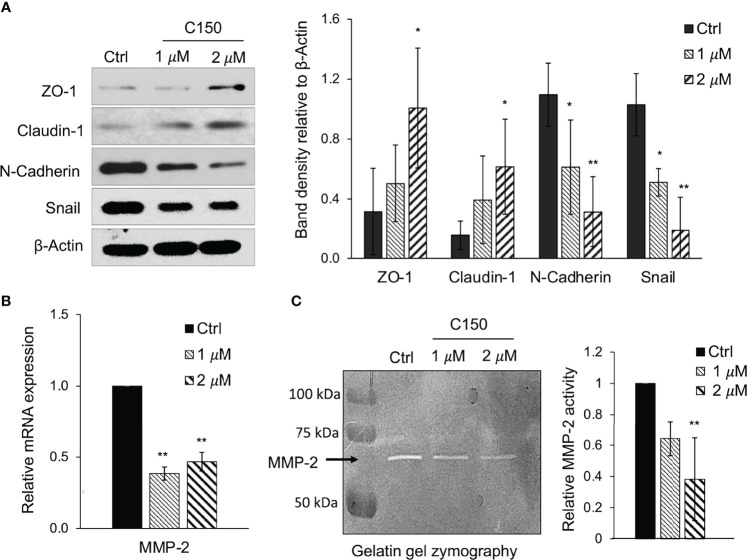
C150 inhibits EMT in PANC-1 cells. **(A)** Western blots of EMT markers. PANC-1 cells were treated with DMSO (Ctrl) or C150 (1 and 2 μM) for 24 h. Total cell lysates were subjected to immunoblotting against ZO-1, claudin-1, Snail and N-cadherin. β-Actin was blotted as loading control. Bar graph shows the band densities relative to β-actin. **(B)** mRNA expression levels of MMP-2. PANC-1 cells were treated with DMSO (Ctrl) or C150 (1 and 2 μM) for 24 h. Reverse transcription-quantitative PCR results were quantified and normalized to the Ctrl group using the 2^-^δδ^Cq^ method with GAPDH as the housekeeping gene. **(C)** MMP-2 activity in gelatin zymography assay. PANC-1 cells were treated with DMSO (Ctrl) or C150 (1 and 2 μM) for 24 h in FBS-free medium. The supernatant was then analyzed in gelatin gel and subjected to enzyme activity zymography assay. Bright bands represent MMP-2 enzyme activity. Bar graph shows the relative enzyme activity normalized to the Ctrl group. Data are presented as mean ± SD of at least three independent experiments in all graphs. ^*^P*<*0.05, ^**^P*<*0.01 (vs. Ctrl) by one-way ANOVA followed by Tukey’s *post hoc* test. C150, 2-[2-(5-nitro-2-thienyl)vinyl]quinoline; Ctrl, control; EMT, epithelial-to-mesenchymal transition; ZO-1, zonula occludens-1.

Multiple EMT markers were examined in order to investigate whether C150 can inhibit this process in pancreatic cancer cells. Upon 24 h of treatment, C150 at 1 and 2 μM significantly increased the expression of the epithelial markers ZO-1 and claudin-1 in PANC-1 cells and decreased the expression of the mesenchymal marker N-cadherin (**Figure 3A**) The levels of the pro-EMT transcription factor Snail also decreased (**Figure 3A**). The metalloprotease MMP-2 had decreased expression at 24 h of treatment with C150 (1 and 2 μM), as detected by RT-qPCR (**Figure 3B**). Accordingly, the activity of MMP-2 was also significantly reduced, as detected by the gelatin zymography assay (**Figure 3C**).

### C150 Decreases Snail Protein Level by Enhancing Its Proteasomal Degradation

**Figure 4:**
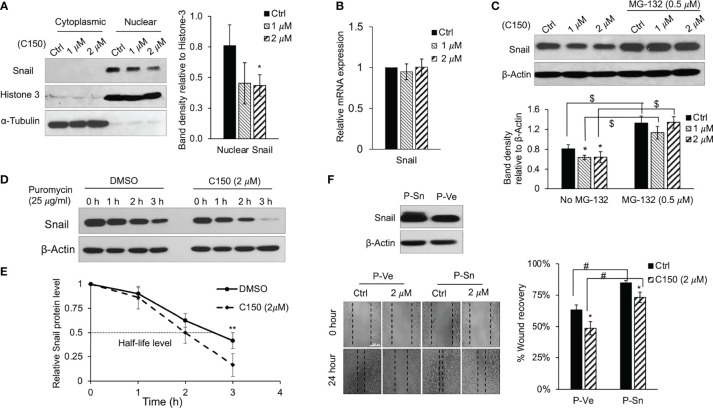
C150 enhances Snail protein degradation. **(A)** Cytoplasmic and nuclear protein levels of Snail. PANC-1 cells were treated with DMSO (Ctrl) or C150 (1 and 2 μM) for 24 h. Cytoplasmic and nuclear fractional lysates were subjected to immunoblotting against Snail. Histone-3 and α-tubulin were blotted as nuclear and cytoplasmic loading controls, respectively. Bar graph shows the band density of nuclear Snail relative to Histone-3. **(B)** mRNA levels of Snail upon C150 treatment. PANC-1 cells were treated with DMSO (Ctrl) or C150 (1 and 2 μM) for 24 h. Reverse transcription-quantitative PCR results were quantified and normalized to the Ctrl group using the 2^-^δδ^Cq^ method with GAPDH as the housekeeping gene. **(C)** Snail protein level upon C150 and MG-132 treatment. PANC-1 cells were treated with C150 (1 and 2 μM) or co-treated with C150 and MG-132 (0.5 μM) for 16 h. Total lysates were subjected to immunoblotting against Snail. β-Actin was blotted as loading control. Bar graph shows the band densities of Snail relative to β-actin. **(D)** Snail protein levels post-puromycin treatment. PANC-1 cells were treated with DMSO or C150 (2 μM) for 24 h, then puromycin (25 μg/ml) was added to block protein synthesis. Cell lysates were collected at 0, 1, 2 or 3 h after puromycin was added. β-Actin was blotted as loading control. **(E)** Snail protein degradation curve. Snail band density relative to β-actin at indicated time points was quantified and normalized to that at 0 h in each group. **(F)** Wound healing assay in PANC-1 cells with Snail overexpression. PANC-1 cells were transfected with Snail plasmid (P-Sn) or empty vector plasmid (P-Ve) for 48 h. Overexpression of Snail was confirmed by western blotting. Transfected cells were then seeded for wound healing assay with DMSO (Ctrl) or C150 (2 μM) treatment for 24 h. Bar graph shows the percentage of wound recovery. Data are presented as mean ± SD of three to five independent experiments. ^*^P*<*0.05, ^**^P*<*0.01 (vs. Ctrl); ^#^P*<*0.05 (P-Sn vs. P-Ve) by **(A–C)** one-way ANOVA followed by Tukey’s *post hoc* test or **(E, F)** Student’s t-test. ^$^P < 0.01 (non-MG-132 vs. MG-132) by Student’s t-test in **(C)**. C150, 2-[2-(5-nitro-2-thienyl)vinyl]quinoline; Ctrl, control.

Snail is a master transcription factor that promotes EMT in pancreatic cancer as well as in several other cancers (26). Accumulation of Snail protein in the nucleus facilitates gene expressions that lead to EMT phenotype. At 24 h of treatment, C150 not only decreased the total Snail protein level (**Figure 3A**), but also significantly decreased the nuclear Snail protein level in PANC-1 cells (**Figure 4A**). The decrease in Snail protein levels may be the result of decreased expression, or enhanced protein degradation. The mRNA expression level of Snail was examined by RT-qPCR and the data demonstrated that the mRNA level was not altered by C150 treatment (**Figure 4B**). Thus, we hypothesized that C150 decreased Snail protein levels by enhancing its proteasomal degradation. PANC-1 cells were treated with C150 in the presence of a proteasome inhibitor, MG-132 (0.5 μM). Treatment with MG-132 completely reversed C150-induced Snail decrease at 16 h (**Figure 4C**). Moreover, when protein synthesis was blocked by puromycin (25 μg/ml) in PANC-1 cells, the presence of C150 (2 μM) accelerated Snail degradation (**Figure 4D**) and shortened the half-life of Snail proteins to 2 h compared to 2.7 h when C150 was absent (**Figure 4E**), supporting the hypothesis that C150 enhances Snail protein degradation.

Snail was then overexpressed in PANC-1 cells. After the overexpression was confirmed by western blotting (**Figure 4F**), cell migration was assessed using wound healing assay. As expected, Snail over expression increased cell migration after 24 h, and C150 treatment (2 μM) inhibited cell migration in both the empty vector and Snail overexpression groups (**Figure 4F**).

Taken together, these *in vitro* data demonstrated that C150 enhanced degradation of the Snail protein, and inhibited pancreatic cancer cell EMT, resulting in inhibition of cell proliferation and cell invasion.

### C150 Treatment Reduces Tumor Growth in an Orthotopic Mouse Model of Pancreatic Cancer

An orthotopic mouse model of pancreatic cancer was used to evaluate the *in vivo* effects of C150. PANC-1-Luc cells were injected into the pancreas of nude mice. Two weeks after cell injection, mice were imaged to confirm tumor formation and were grouped based on tumor burden into vehicle (n=10) and treatment group (n=9), for each group to have equal average tumor burdens. Mice were treated 3 times per week with 15 mg/kg of C150 (determined by the dose-finding experiments described in Materials and methods) or vehicle (20% DMSO + 20% H_2_O + 60% PEG400) by IP injection.

**Figure 5:**
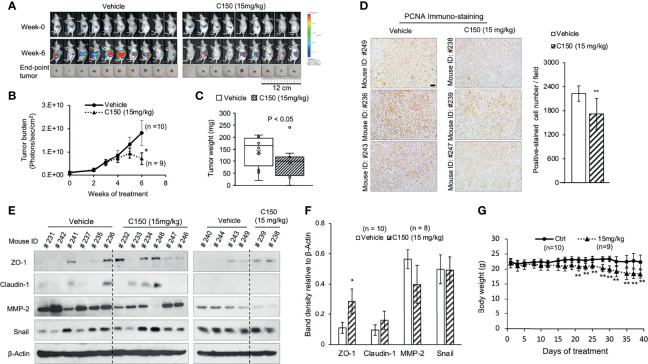
C150 reduces tumor growth in mice. **(A)** Bioluminescence images of tumor burden in live mice at the beginning (week 0) and at the end (week 6) of treatments. Actual tumors at sacrifice were shown below the bioluminescence images. **(B)** Longitudinal mean tumor burden detected by imaging, quantified as photons/sec/cm^2^ (mean ± SEM). **(C)** Tumor weight at sacrifice (n=9 for the C150 group and n=10 for the vehicle group). ^*^P*<*0.05 by Mann-Whitney U test (vs. vehicle control). **(D)** Immunohistochemistry staining for PCNA in tumor samples. Three random tumors from each of the vehicle or C150 groups were analyzed. Scale bar, 50 μm. Bar graph shows the quantification of positively stained cells, presented as mean ± SD of at least five different fields per sample of the three samples. ^**^P*<*0.01 with Student’s t-test (vs. vehicle control). **(E)** Western blot analysis of epithelial-to-mesenchymal transition markers in tumor samples. Total lysates of tumor samples from all mice were analyzed. β-Actin was blotted as loading control. The tumor samples were collected 48 hours after the last treatment (mice were treated for 6 weeks, 3 times a week). **(F)** Quantification of western blot band density relative to β-actin (mean ± SEM). (*, P = 0.05 vs. Vehicle). **(G)** Mouse body weight (mean ± SD). ^**^P*<*0.01 by Student’s t-test (vs. vehicle control). C150, 2-[2-(5-nitro-2-thienyl)vinyl]quinoline; Ctrl, control; PCNA, proliferating cell nuclear antigen.

Six weeks of C150 treatment significantly reduced tumor burden compared to vehicle controls (**Figures 5A, B**). The final tumor weight in the C150-treated group was significantly lower compared with that in the vehicle-treated group (**Figure 5C**). Tumor samples were collected 48 hours after the last treatment and evaluated by immunoblots. PCNA was significantly inhibited in C150-treated tumors compared with vehicle-treated tumors (**Figure 5D**), indicating the reduction in cell proliferation by C150 treatment. EMT markers in the tumor samples were examined by western blotting. Consistent with the *in vitro* data, the levels of the epithelial markers ZO-1 was elevated (P = 0.05 vs. vehicle). Claudin-1 showed a trend to elevation in the tumor tissues of C150-treated mice but the change was not statistically significant, whereas the pro-metastasis marker MMP-2 trended to decrease with C150 treatment (**Figures 5E, F**). Collectively, the *in vivo* data were consistent with the *in vitro* data, indicating that C150 inhibits pancreatic cancer progression.

Toxicities were evaluated base on BCS and clinical signs, as described in Materials and methods. All mice started at a BCS of 3. During treatment, no mice exhibited observable clinical signs of toxicity, or had BCS drop to 2 or <2. However, the mean body weight of the treatment group decreased compared with that of the control group (**Figure 5G**), indicating potential toxicities not documented herein. Therefore, the toxicity profile and therapeutic window of C150 require further investigation.

## Discussion

Accumulating evidence has demonstrated the critical role of EMT in promoting pancreatic cancer progression (9). During EMT, cancer cells undergo serial molecular changes during which they lose epithelial markers and acquire mesenchymal markers, leading to the loss of cell-cell and cell-membrane adhesion (27). In this study, C150 treatment resulted in increased expression of the epithelial markers ZO-1 and claudin-1 in pancreatic cancer cells, both of which are integral components of tight junctions in cells with an epithelial phenotype. In addition, the expression of the mesenchymal marker N-cadherin was decreased by C150 treatment. These data indicated that C150 treatment suppressed the EMT process in PANC-1 cells. These mechanisms were further confirmed in a mouse model.

During invasion, cancer cells degrade the basement membrane and the dense ECM. This degradation process is mainly mediated by the MMPs (28). The production of MMPs by cancer cells is upregulated by EMT-promoting factors (10, 29). In the present study, compound C150 significantly inhibited the activity of MMP-2, which is one of the main enzymes responsible for degrading collagen fibers in the tumor stroma. This may be of particular importance in pancreatic cancer, as the disease is characterized by rich collagenous stroma (30). An 3-D cell invasion assay with collagenous stroma here showed the outcome of reduced pancreatic cancer cell invasion with C150 treatment.

The levels of the pro-EMT factor Snail were markedly decreased by C150 treatment. Snail is a master transcription factor that induces EMT in a number of cancer types (26, 31). Snail directly regulates the expression of several EMT markers, including ZO-1, claudin-1 and N-cadherin (12, 17, 18). Ectopic expression of Snail resulted in EMT and promoted metastasis in mouse models of pancreatic cancer (22, 32). The reduction in Snail protein levels by C150 treatment indicates a mechanism through which C150 inhibits EMT in pancreatic cancer cells. Furthermore, silencing Snail was reported to result in cell cycle arrest and suppressed proliferation in cancer (33). This observation is consistent with our data indicating that compound C150 decreased Snail expression and inhibited cell proliferation. A limitation of the present study is that changes in Snail expression were not detected in the mouse tumor samples. This is likely due to several reasons: The fast turnover time of the Snail protein (34, 35) and a late sample collection at 48 h after the last treatment may lead to changes in Snail protein levels remaining undetected. Furthermore, tissues harvested from mouse tumors were a mixture of tumor cells, fibroblasts, blood vessels, immune cells and other associated tissue components, which may complicate the interpretation of the results. In addition, after 6 weeks of treatment, the tumor cells exhibiting decreased Snail expression (hypothesized to be more sensitive) would have become dormant. Therefore, in the residual tumor tissues, cells without or with subtler changes in Snail expression are more likely to become the dominant population, and a Snail decrease cannot be detected in the residual tumor. Nevertheless, multiple *in vitro* assays and Snail overexpression assays confirmed the role of Snail in C150-mediated EMT-inhibiting effect.

Of note, C150 induced a decrease in Snail levels through enhanced proteasomal degradation, but not through inhibiting its expression, as Snail mRNA remained unchanged after C150 treatment. Snail protein degradation is tightly controlled by its phosphorylation and ubiquitination (36, 37). Multiple pathways have been reported to regulate Snail phosphorylation, among which is the GSK3β mediated pathway (38). GSK3β phosphorylates Snail at two different sites, priming it for nuclear exportation, ubiquitination and subsequent proteasomal degradation (38). However, our data showed that C150 significantly increased Ser9 phosphorylation of GSK3β (**Supplementary Digital Content 1**), thereby inhibiting its kinase function (39, 40). Therefore, Ser9 phosphorylation of GSK3β would likely increase Snail protein stability rather than decrease it. Thus, it was inferred that the C150-induced Snail degradation is likely not mediated by the GSK3β pathway. A number of regulatory pathways and kinases are involved in the regulation of Snail protein stability (12, 36). As the molecular target of C150 remains unknown, the exact mechanisms through which C150 promotes Snail proteasomal degradation are unclear and require further investigation.

The high heterogenicity of pancreatic cancer may influence C150 treatment outcome and contribute to insensitivity/resistance of some tumors to C150 treatment. Some tumors may have cells with upregulated efflux transporters upon drug treatment, and/or harbor mutations in the targeted genes/pathways that dampened the treatment effects. Some tumors may have different tumor stroma densities and compositions that influence drug penetration to reach the cancer cells and/or responses of the cells to the treatment. While the data here showed overall efficacy, the single-drug treatment of C150 had in some of the mice only a modest decrease in tumor burden. This argues for a combination treatment targeting different pathways for better outcomes.

To the best of our knowledge, the present study was the first to report that the novel compound C150 inhibits pancreatic cancer cell migration and invasion *in vitro*, suppress EMT and reduce tumor growth in mice. The data suggested that C150 may serve as a drug lead for comprehensive inhibition of pancreatic cancer growth and metastasis. Future investigation should focus on identification of the target of C150 and in-depth mechanistic studies, as well as determination of its toxicity profiles. Analogues of C150 may be developed and tested for improved efficacy, reduced toxicity and improved pharmacokinetics and other drug-like properties.

## Data Availability Statement

The original contributions presented in the study are included in the article/**Supplementary Material**. Further inquiries can be directed to the corresponding author.

## Ethics Statement

The animal study was reviewed and approved by The Institutional Animal Care and Use Committee of the University of Kansas Medical Center.

## Author Contributions

QC conceptualized and oversaw the studies and provided resources. QC and TW designed the experiments. TW performed the experiments, interpreted the data and wrote the manuscript. PC assisted with data collection, analysis and discussion. MB and SW performed the compound solubility test and participated in data interpretation. RD and FS participated in data analysis and discussion. QC, TW, PC, RD, and FS have seen and can confirm the authenticity of the raw data. All authors contributed to the article and approved the submitted version.

## Funding

The present study was supported by a Lied Basic Science Pilot Project Award through the Frontiers Pilot and Collaborative Funding Program provided by the NIH/NCATS, and partially by a bridging grant from the University of Kansas Research Institute (Kansas, USA), and a grant provided by the GR’s Foundation, Mosby Lincoln Foundation, Donlan Foundation (Kansas, USA) and the University of Kansas Cancer Center support grant (P30 CA168524).

## Conflict of Interest

The authors declare that the research was conducted in the absence of any commercial or financial relationships that could be construed as a potential conflict of interest.

## Publisher’s Note

All claims expressed in this article are solely those of the authors and do not necessarily represent those of their affiliated organizations, or those of the publisher, the editors and the reviewers. Any product that may be evaluated in this article, or claim that may be made by its manufacturer, is not guaranteed or endorsed by the publisher.

## References

[1] (IARC) IAfRoC. World Cancer Report. In: Bernard W, editor. World Cancer Report 2014. Lyon Cedex, France: Steward CPW (2014).

[2] Siegel RL Miller KD Jemal A. Cancer Statistics, 2019. CA: A Cancer J Clin (2019) 69:7–34. doi: 10.3322/caac.21551 30620402

[3] Burris HA 3rd Moore MJ Andersen J Green MR Rothenberg ML Modiano MR. Improvements in Survival and Clinical Benefit With Gemcitabine as First-Line Therapy for Patients With Advanced Pancreas Cancer: A Randomized Trial. J Clin Oncol (1997) 15:2403–13. doi: 10.1200/JCO.1997.15.6.2403 9196156

[4] Von Hoff DD Ervin T Arena FP Chiorean EG Infante J Moore M. Increased Survival in Pancreatic Cancer With Nab-Paclitaxel Plus Gemcitabine. N Engl J Of Med (2013) 369:1691–703. doi: 10.1056/NEJMoa1304369 PMC4631139 24131140

[5] Conroy T Desseigne F Ychou M Bouche O Guimbaud R Becouarn Y. FOLFIRINOX Versus Gemcitabine for Metastatic Pancreatic Cancer. N Engl J Of Med (2011) 364:1817–25. doi: 10.1056/NEJMoa1011923 21561347

[6] Kamisawa T Wood LD Itoi T Takaori K. Pancreatic Cancer. Lancet (2016) 388:73–85. doi: 10.1016/S0140-6736(16)00141-0 26830752

[7] Loos M Kleeff J Friess H Buchler MW. Surgical Treatment of Pancreatic Cancer. Ann New York Acad Sci (2008) 1138:169–80. doi: 10.1196/annals.1414.024 18837898

[8] Wang S Huang S Sun YL. Epithelial-Mesenchymal Transition in Pancreatic Cancer: A Review. BioMed Res Int (2017) 2017:10. doi: 10.1155/2017/2646148 PMC5742883 29379795

[9] Beuran M Negoi I Paun S Ion AD Bleotu C Negoi RI. The Epithelial to Mesenchymal Transition in Pancreatic Cancer: A Systematic Review. Pancreatology (2015) 15:217–25. doi: 10.1016/j.pan.2015.02.011 25794655

[10] Tsai JH Yang J. Epithelial-Mesenchymal Plasticity in Carcinoma Metastasis. Genes Dev (2013) 27:2192–206. doi: 10.1101/gad.225334.113 PMC3814640 24142872

[11] Thiery JP. Epithelial–Mesenchymal Transitions in Tumour Progression. Nat Rev Cancer (2002) 2:442–54. doi: 10.1038/nrc822 12189386

[12] Lamouille S Xu J Derynck R. Molecular Mechanisms of Epithelial–Mesenchymal Transition. Nat Rev Mol Cell Biol (2014) 15:178. doi: 10.1038/nrm3758 24556840 PMC4240281

[13] Kalluri R Weinberg RA. The Basics of Epithelial-Mesenchymal Transition. J Clin Invest (2009) 119:1420–8. doi: 10.1172/JCI39104 PMC2689101 19487818

[14] Rhim Andrew D Mirek Emily T Aiello Nicole M Maitra A Bailey Jennifer M McAllister F. EMT and Dissemination Precede Pancreatic Tumor Formation. Cell (2012) 148:349–61. doi: 10.1016/j.cell.2011.11.025 PMC3266542 22265420

[15] Hotz B Arndt M Dullat S Bhargava S Buhr H-J Hotz HG. Epithelial to Mesenchymal Transition: Expression of the Regulators Snail, Slug, and Twist in Pancreatic Cancer. Clin Cancer Res (2007) 13:4769–76. doi: 10.1158/1078-0432.CCR-06-2926 17699854

[16] Batlle E Sancho E Franci C Dominguez D Monfar M Baulida J. The Transcription Factor Snail Is a Repressor of E-Cadherin Gene Expression in Epithelial Tumour Cells. Nat Cell Biol (2000) 2:84–9. doi: 10.1038/35000034 10655587

[17] Ikenouchi J Matsuda M Furuse M Tsukita S. Regulation of Tight Junctions During the Epithelium-Mesenchyme Transition: Direct Repression of the Gene Expression of Claudins/Occludin by Snail. J Cell Sci (2003) 116:1959–67. doi: 10.1242/jcs.00389 12668723

[18] Ohkubo T Ozawa M. The Transcription Factor Snail Downregulates the Tight Junction Components Independently of E-Cadherin Downregulation. J Cell Sci (2004) 117:1675–85. doi: 10.1242/jcs.01004 15075229

[19] Jorda M Olmeda D Vinyals A Valero E Cubillo E Llorens A. Upregulation of MMP-9 in MDCK Epithelial Cell Line in Response to Expression of the Snail Transcription Factor. J Cell Sci (2005) 118:3371–85. doi: 10.1242/jcs.02465 16079281

[20] Shields MA Dangi-Garimella S Krantz SB Bentrem DJ Munshi HG. Pancreatic Cancer Cells Respond to Type I Collagen by Inducing Snail Expression to Promote Membrane Type 1 Matrix Metalloproteinase-Dependent Collagen Invasion. J Biol Chem (2011) 286:10495–504. doi: 10.1074/jbc.M110.195628 PMC3060503 21288898

[21] Yokoyama K Kamata N Fujimoto R Tsutsumi S Tomonari M Taki M. Increased Invasion and Matrix Metalloproteinase-2 Expression by Snail-Induced Mesenchymal Transition in Squamous Cell Carcinomas. Int J Oncol (2003) 22:891–8. doi: 10.3892/ijo.22.4.891 12632084

[22] Nishioka R Itoh S Gui T Gai Z Oikawa K Kawai M. SNAIL Induces Epithelial-to-Mesenchymal Transition in a Human Pancreatic Cancer Cell Line (BxPC3) and Promotes Distant Metastasis and Invasiveness *In Vivo* . Exp Mol Pathol (2010) 89:149–57. doi: 10.1016/j.yexmp.2010.05.008 20576520

[23] Polireddy K Dong R McDonald PR Wang T Luke B Chen P. Targeting Epithelial-Mesenchymal Transition for Identification of Inhibitors for Pancreatic Cancer Cell Invasion and Tumor Spheres Formation. PloS One (2016) 11:e0164811. doi: 10.1371/journal.pone.0164811 27764163 PMC5072586

[24] Livak KJ Schmittgen TD. Analysis of Relative Gene Expression Data Using Real-Time Quantitative PCR and the 2(-Delta Delta C(T)) Method. Methods (2001) 25:402–8. doi: 10.1006/meth.2001.1262 11846609

[25] Sumi T Matsumoto K Takai Y Nakamura T. Cofilin Phosphorylation and Actin Cytoskeletal Dynamics Regulated by Rho- and Cdc42-Activated LIM-Kinase 2. J Cell Biol (1999) 147:1519–32. doi: 10.1083/jcb.147.7.1519 PMC2174243 10613909

[26] Nieto MA. The Snail Superfamily of Zinc-Finger Transcription Factors. Nat Rev Mol Cell Biol (2002) 3:155–66. doi: 10.1038/nrm757 11994736

[27] Ye X Weinberg RA. Epithelial-Mesenchymal Plasticity: A Central Regulator of Cancer Progression. Trends Cell Biol (2015) 25:675–86. doi: 10.1016/j.tcb.2015.07.012 PMC4628843 26437589

[28] Jablonska-Trypuc A Matejczyk M Rosochacki S. Matrix Metalloproteinases (MMPs), the Main Extracellular Matrix (ECM) Enzymes in Collagen Degradation, as a Target for Anticancer Drugs. J Enzyme Inhib Med Chem (2016) 31:177–83. doi: 10.3109/14756366.2016.1161620 27028474

[29] Gordon KJ Kirkbride KC How T Blobe GC. Bone Morphogenetic Proteins Induce Pancreatic Cancer Cell Invasiveness Through a Smad1-Dependent Mechanism That Involves Matrix Metalloproteinase-2. Carcinogenesis (2009) 30:238–48. doi: 10.1093/carcin/bgn274 PMC2639045 19056927

[30] Hidalgo M. Pancreatic Cancer. N Engl J Med (2010) 362:1605–17. doi: 10.1056/NEJMra0901557 20427809

[31] Wang Y Shi J Chai K Ying X Zhou BP. The Role of Snail in EMT and Tumorigenesis. Curr Cancer Drug Targets (2013) 13(9):963–72. doi: 10.2174/15680096113136660102 PMC4004763 24168186

[32] Yin T Wang C Liu T Zhao G Zha Y Yang M. Expression of Snail in Pancreatic Cancer Promotes Metastasis and Chemoresistance. J Surg Res (2007) 141:196–203. doi: 10.1016/j.jss.2006.09.027 17583745

[33] Yang X Han M Han H Wang B Li S Zhang Z. Silencing Snail Suppresses Tumor Cell Proliferation and Invasion by Reversing Epithelial-to-Mesenchymal Transition and Arresting G2/M Phase in Non-Small Cell Lung Cancer. Int J Oncol (2017) 50:1251–60. doi: 10.3892/ijo.2017.3888 28259904

[34] de Herreros AG Peiró S Nassour M Savagner P. Snail Family Regulation and Epithelial Mesenchymal Transitions in Breast Cancer Progression. J Mammary Gland Biol Neoplasia (2010) 15:135–47. doi: 10.1007/s10911-010-9179-8 PMC2930904 20455012

[35] Zheng H Shen M Zha YL Li W Wei Y Blanco MA. PKD1 Phosphorylation-Dependent Degradation of SNAIL by SCF-FBXO11 Regulates Epithelial-Mesenchymal Transition and Metastasis. Cancer Cell (2014) 26(3):358–73. doi: 10.1016/j.ccr.2014.07.022 PMC4159622 25203322

[36] Yu Q Zhou BP Wu Y. The Regulation of Snail: On the Ubiquitin Edge. Cancer Cell Microenviron (2017) 4(2):e1567. 29147673 PMC5685547

[37] Díaz VM Viñas-Castells R García de Herreros A. Regulation of the Protein Stability of EMT Transcription Factors. Cell Adh Migr (2014) 8:418–28. doi: 10.4161/19336918.2014.969998 PMC4594480 25482633

[38] Zhou BP Deng J Xia W Xu J Li YM Gunduz M. Dual Regulation of Snail by GSK-3β-Mediated Phosphorylation in Control of Epithelial–Mesenchymal Transition. Nat Cell Biol (2004) 6:931–40. doi: 10.1038/ncb1173 15448698

[39] Frame S Cohen P Biondi RM. A Common Phosphate Binding Site Explains the Unique Substrate Specificity of GSK3 and Its Inactivation by Phosphorylation. Mol Cell (2001) 7:1321–7. doi: 10.1016/S1097-2765(01)00253-2 11430833

[40] Hermida MA Dinesh Kumar J Leslie NR. GSK3 and its Interactions With the PI3K/AKT/mTOR Signalling Network. Adv Biol Regul (2017) 65:5–15. doi: 10.1016/j.jbior.2017.06.003 28712664

