# Supplementary material for: Novel Compound C150 Inhibits Pancreatic Cancer Cell Epithelial-to-Mesenchymal Transition and Tumor Growth in Mice
Source: Front Oncol. 2021 Dec 15;11:773350. doi: 10.3389/fonc.2021.773350 (PMC8714879; doi:10.3389/fonc.2021.773350)
Supplement: Supplementary file 1 [file DataSheet_1.pdf]

Supplementary Digital Content 1.

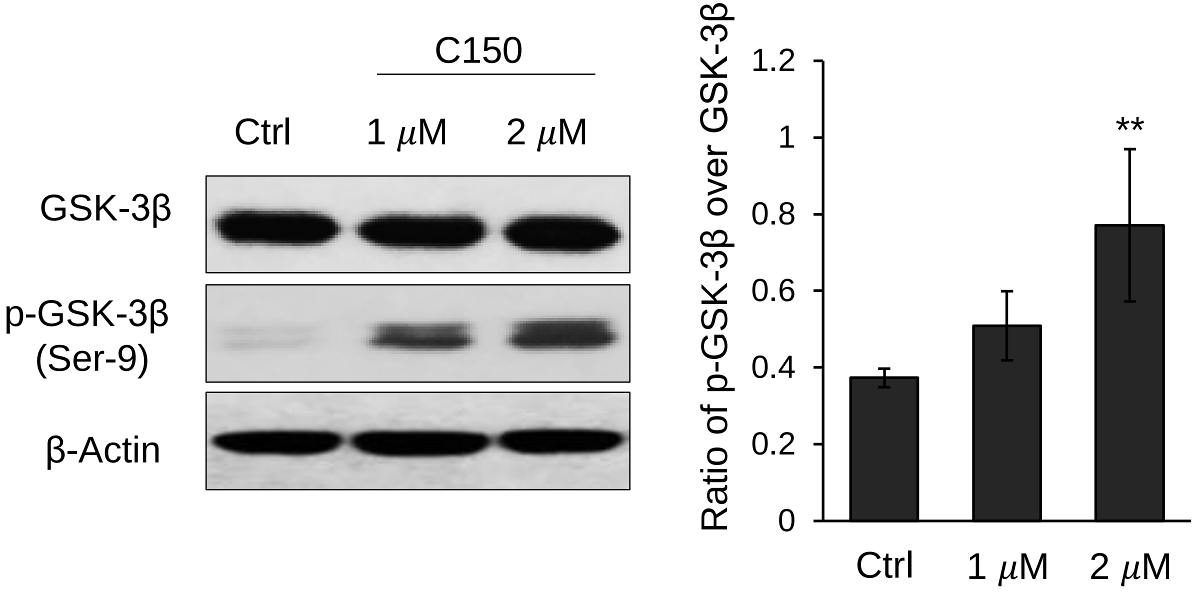

Supplementary Digital Content 1. C150 treatment increased Serine-9 phosphorylation of GSK-3β. PANC-1 cells were treated with DMSO (Ctrl) or C150 at 1 μM and 2 μM for 24 hours. Total cell lysate was subjected to immunoblotting analysis against GSK-3β and p-GSK-3β (Ser-9). β-Actin was blotted as loading control. Right panel bar graph shows the ratio of band density of p-GSK-3β (Ser-9) over GSK-3β. Data presented as Mean ±SD of 3 independent experiments. ** $p < 0.01$ (vs. Ctrl) by One-way ANOVA-Tukey's test.
